# Resolution of Sigmoid Esophagus After Laparoscopic Adjustable Gastric Band Removal

**DOI:** 10.7759/cureus.67139

**Published:** 2024-08-18

**Authors:** Ty Bayliss, Yosuke Sakurai, Pranav Balakrishnan, Abigail Murphy, Semeret Munie

**Affiliations:** 1 General Surgery, Marshall University Joan C. Edwards School of Medicine, Huntington, USA; 2 Bariatric Surgery, Marshall University Joan C. Edwards School of Medicine, Huntington, USA

**Keywords:** bariatric surgery, alimentary tract, pseudoachalasia, sigmoid esophagus, laparoscopic adjustable gastric band

## Abstract

Pseudoachalasia is a known complication following a gastric band placement that is reversible with band removal. However, the development of a sigmoid esophagus is uncommon. Sigmoid esophagus is considered late-stage achalasia and is associated with worse outcomes with myotomy compared to earlier-stage achalasia. A 53-year-old male with a laparoscopic adjustable gastric band (LAGB) placed 15 years ago presented to the clinic with persistent dysphagia after the band was deflated for symptoms of dysphagia. The upper gastrointestinal series showed a 6.6-cm-diameter, tortuous, sigmoid-shaped esophagus. Esophagogastroduodenoscopy confirmed a diagnosis of sigmoid esophagus proven via the presence of inflamed mucosa, tortuous esophagus, and high lower esophagus sphincter pressure consistent with pseudoachalasia, all secondary to LAGB. The patient then underwent band removal, resulting in rapid resolution of his symptoms. The postoperative barium study showed improvement in dilatation. At the three-month postoperative follow-up, manometry demonstrated normal motility, indicating resolution of the pseudoachalasia and sigmoid esophagus. This case demonstrated band removal as an effective treatment option despite late-stage pseudoachalasia with a sigmoid esophagus.

## Introduction

Pseudoachalasia is a known complication following gastric band placement that is reversible with band removal [1]. However, the development of a sigmoid esophagus is uncommon. In patients with achalasia, sigmoid esophagus is considered late-stage achalasia and is associated with worse outcomes compared to earlier-stage achalasia [2]. Herein, we present an article that was presented at the 2023 American Society for Metabolic and Bariatric Surgery (ASMBS) Meeting in Las Vegas, NV, describing a patient who developed a sigmoid esophagus secondary to gastric band placement that was resolved with the removal of the band.

## Case presentation

This is a case report of a 53-year-old male with a history of laparoscopic adjustable gastric banding (LAGB) 15 years prior who presented to the clinic due to persistent dysphagia over the years after band placement. At the time of placement of the LABG, the patient weighed 129 kg, BMI 40, and at the time of re-presentation, he weighed 123 kg, BMI 39. Although he has had dysphagia for years, he did not seek any medical attention until noticeably worsening dysphagia a couple of months prior to presentation brought the patient to the clinic.

**Figure 1:**
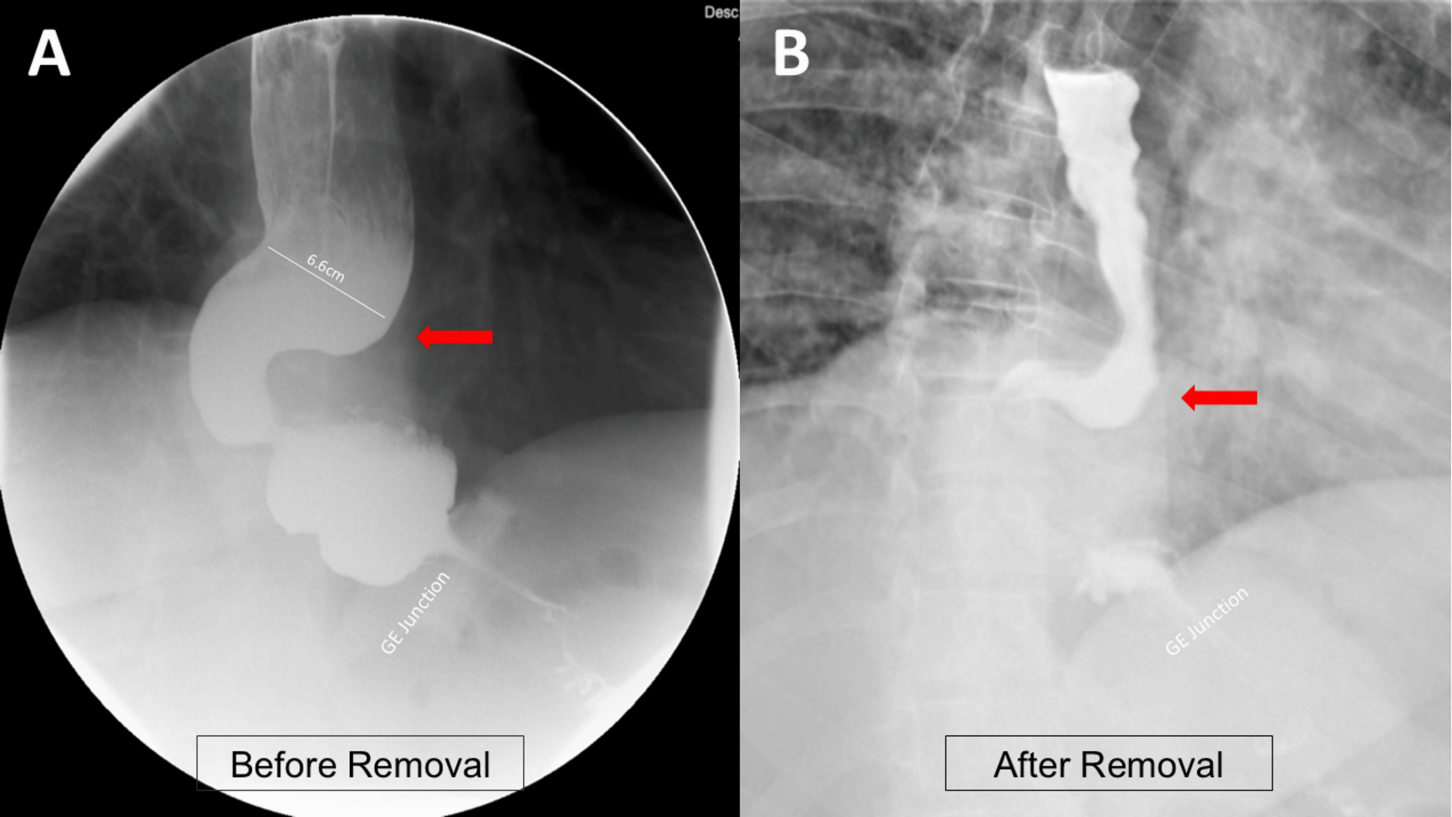
Pre- and postoperative barium swallow This figure demonstrates how the patient's esophagus changed from (A) before the laparoscopic adjustable gastric band (LABG) removal to (B) after removal. The patient's esophagus became less dilated, and the tortuous shape had resolved.

**Figure 2:**
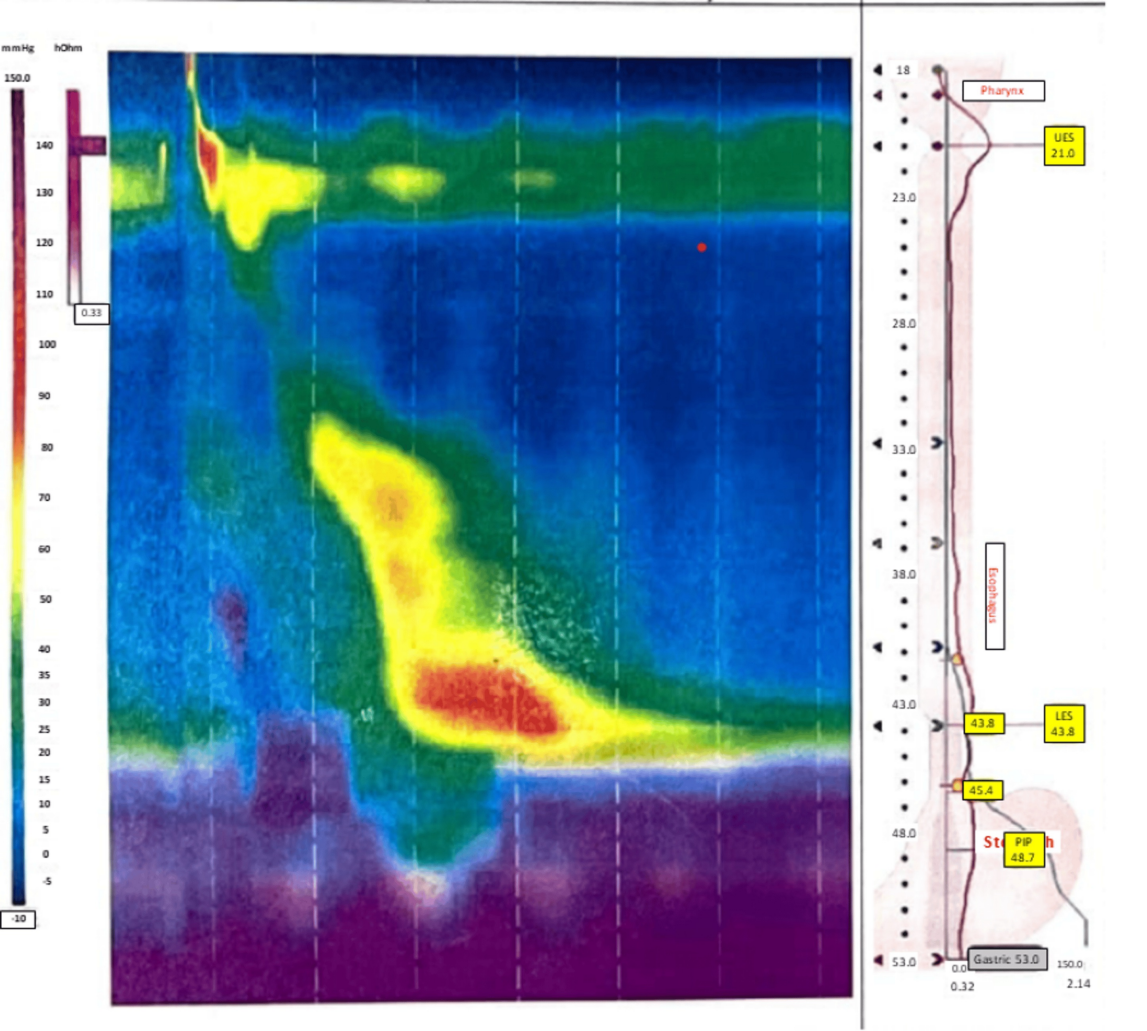
Post-band removal manometry The manometry report after the laparoscopic adjustable gastric band (LAGB) removal shows normal esophageal pressures down to the lower esophageal sphincter.

In the clinic, the band was deflated, but the patient continued to experience dysphagia with nausea and vomiting. Upon further investigation, the patient underwent an upper gastrointestinal series that demonstrated a dilated, tortuous sigmoid esophagus measuring 6.6 cm in diameter (Figure 1A). An esophagogastroduodenoscopy was performed, which revealed an inflamed mucosa, a tortuous esophagus, and a high lower esophagus sphincter pressure. A biopsy of the distal esophagus was also performed and did not show any evidence of malignancy. These findings were consistent with a diagnosis of pseudoachalasia secondary to LAGB, resulting in a sigmoid esophagus. The patient subsequently underwent robotic-assisted gastric band removal, which resulted in rapid resolution of symptoms over the patient's postoperative course. On postoperative day one, the patient underwent a barium study showing improvement of the esophageal dilatation (Figure 1B). The patient tolerated a liquid diet and was discharged later on postoperative day one. At the three-month postoperative follow-up, the patient reported tolerating a regular diet, and high-resolution manometry demonstrated normal motility (Figure 2).

## Discussion

Pseudoachalasia is a clinical condition mimicking idiopathic achalasia, most commonly associated with malignancy at the gastroesophageal junction [3]. Our patient did not have any evidence of malignancy. Although pseudoachalasia following a gastric band is a recognized complication, its reported incidence was previously documented at 1.9% [1]. The time interval between the band placement and the development of pseudoachalasia varies in studies, ranging from 8 to 32 months [1,3]. Notably, previous studies suggested that males were rarely affected by pseudoachalasia following LAGB [3]. We suspect this specific case of pseudoachalasia occurred due to increased outflow resistance from LAGB placement.

The duration of this patient's symptoms probably led to the patient developing a sigmoid esophagus, which is an unusual presentation. Sigmoid esophagus is the term used to describe when the esophagus takes on a dilated, sigmoidal shape, typically arising during late-stage achalasia. Although pseudoachalasia following LAGB is generally reversible with band deflation, the late stage of pseudoachalasia may necessitate band removal, with the potential for conversion to Roux-en-Y gastric bypass or sleeve gastrectomy [1,4]. This patient's clinical course emphasizes the importance of ruling out malignancy, recognizing complications following LAGB, and maintaining long-term follow-up after bariatric surgery.

Treatment of a typical case of sigmoid esophagus poses unique challenges due to the resistance to various interventions such as pneumatic dilation, botulinum toxin injection, or myotomy among patients with achalasia. While esophagectomy is the definitive treatment, it carries a 5.4% mortality rate when specifically employed for the treatment of sigmoid esophagus [2]. In our case, the patient presented with pseudoachalasia, making these previously described conventional interventions less desirable. Simple adjustments such as balloon deflation failed to alleviate symptoms; however, band removal led to the rapid resolution of symptoms. Postoperative manometry revealed normal motility, supporting the resolution of pseudoachalasia despite his late presentation. This case suggests that even in the late-stage presentation of pseudoachalasia, band removal remains an effective treatment option. Long-term follow-up is often necessary to monitor weight regain and band placement complications such as dysphagia, esophageal or port site erosion, and band displacement. Many of these can be treated with band removal or alternative surgical or medical interventions to correct the problem.

## Conclusions

This case highlights sigmoid esophagus as a potential complication of gastric banding and as a late presentation of pseudoachalasia. Despite the challenges of managing sigmoid esophagus, band removal may serve as an effective treatment option. Over the months following band removal, the patient's sigmoid esophagus resolved. This case underscores the need for long-term follow-up following bariatric surgery and recognition of the complications. Long-term follow-up ensures that any potential complications have time to develop and be recognized by the medical care team.
